# MxB Disrupts Hepatitis C Virus NS5A–CypA Complex: Insights From a Combined Theoretical and Experimental Approach

**DOI:** 10.3389/fmicb.2022.849084

**Published:** 2022-03-17

**Authors:** Quanjie Li, Ni An, Xiao Yin, Ruixin Zhang, Huihan Shao, Dongrong Yi, Shan Cen

**Affiliations:** ^1^Department of Immunology, Institute of Medicinal Biotechnology, Chinese Academy of Medical Sciences, Beijing, China; ^2^Center for Drug Evaluation, National Medical Products Administration, Beijing, China

**Keywords:** MxB, NS5A, CypA, protein-protein interaction, host antiviral innate immunity, HCV

## Abstract

The human myxovirus resistance B (MxB) protein is an interferon-induced restriction factor that fights a wide range of viruses. We previously demonstrated that MxB binds to hepatitis C virus (HCV)-encoded non-structural protein 5A (NS5A) and inhibits HCV infection by impairing the formation of cyclophilin A (CypA)–NS5A complex. However, the molecular details about how the presence of MxB diminishes the binding of NS5A to CypA remain uncovered. In this study, through molecular dynamic simulations and biochemical assays, we characterized that MxB binds to NS5A domain I through its N-terminal and GTPase domains. Specifically, amino acids (aa.) 189–191 and aa. 330–334 within MxB, together with NS5A residues aa. 71–73, are crucial for MxB–NS5A interaction. Furthermore, we predicted the CypA:NS5A and CypA:NS5A:MxB complexes and calculated the per-residue energy decomposition for identified key residues of the CypA–NS5A interface. A 28% decrease in CypA–NS5A binding affinity was observed in the presence of MxB, suggesting a weakened CypA–NS5A association upon binding of MxB to NS5A, which may contribute to the MxB-mediated inhibitory effect on the formation of CypA–NS5A complex. This work provides information for the antiviral mechanism of MxB and may facilitate the discovery of new strategies to combat CypA-dependent viruses.

## Introduction

The human myxovirus resistance B (MxB) protein is a member of the dynamin-like large guanosine triphosphatases (GTPases) family (Alvarez et al., 2017). It has previously been described as an interferon-induced restriction factor of HIV-1 (Goujon et al., 2013; Kane et al., 2013; Liu et al., 2013; Wang et al., 2020), herpesviruses (Crameri et al., 2018), and influenza A virus (Steiner and Pavlovic, 2020). In 2019, we, for the first time, reported that MxB significantly inhibits flaviviruses, especially hepatitis C virus (HCV), in a cyclophilin A (CypA)-dependent manner (Yi et al., 2019). Mounting evidence indicates that the host cell factor CypA is indispensable for HCV replication (Yang et al., 2008; Chatterji et al., 2009; Liu et al., 2009), and the binding of CypA to HCV-encoded non-structural protein NS5A promotes viral RNA replication (Hanoulle et al., 2009; Chatterji et al., 2010; Waller et al., 2010; Yang et al., 2010; Foster et al., 2011; Verdegem et al., 2011). Cyclophilin inhibitors that disrupt CypA–NS5A interaction would inhibit HCV replication (Hopkins et al., 2012). Interestingly, MxB exhibited its anti-HCV activity in a similar manner. Data in our previous study revealed that MxB binds directly to NS5A, thereby impairing NS5A interaction with CypA (Yi et al., 2019). However, the structural insights into the inhibitory mechanisms of MxB at an atomistic level remain fully unsolved.

The crystal structure of MxB (PDB ID 4WHJ) revealed three functional domains: the GTPases domain that is responsible for GTP binding and hydrolysis, the stalk domain that is critical for oligomerization, and a bundle signaling element (BSE) domain that connects GTPases and stalk domains (Fribourgh et al., 2014). It should be noted that the N-terminal 83-aa of MxB, another function domain that is crucial for its antivirus activity (Kane et al., 2013; Lemke et al., 2013; Fribourgh et al., 2014; Goujon et al., 2014; Yi et al., 2019), is predicted to be unstructured and could not be crystallized. We had previously reported that MxB was specifically associated with NS5A and did not interact with other HCV proteins. As a multifunctional protein, NS5A is involved in HCV replicase through interacting with various viral and host factors (Shi et al., 2002; He et al., 2006). Recent studies suggested that NS5A comprises an N-terminal membrane anchor and three domains (I, II, and III) (Tellinghuisen et al., 2004). A later work by us affirmed that domain I (NS5A-D1) is responsible for MxB–NS5A interaction (Yi et al., 2019). Despite this progress, the detailed molecular features of the MxB–NS5A binding interface remain elusive.

Unlike MxB, which binds to NS5A-D1, CypA interacts with NS5A-D2D3, and the interaction between CypA and NS5A has been extensively studied (Hanoulle et al., 2009; Foster et al., 2011; Verdegem et al., 2011; Ngure et al., 2016). Grise et al. (2012) pointed out that CypA bind to the proline-rich region of NS5A-D2D3, and two specific proline residues (P310 and P341) of NS5A were required for CypA function. Ngure et al. (2016) showed that W316 of NS5A is essential for the interaction with CypA. Verdegem et al. (2011) reported that CypA interacts with NS5A-D3, and this interaction is completely abolished by cyclosporin A. Besides, CypA mutants R55A, F60A, F113A, and H126Q drastically or completely abolished NS5A binding (Yang et al., 2010; Foster et al., 2011). These mutation studies provide valuable information for the prediction of the CypA–NS5A complex structure. To date, the X-ray structure of CypA has been elucidated (PDB ID 1YND) (Kallen et al., 2005), while the structural information on the intrinsically disordered NS5A-D2D3 has been limited.

To figure out the aforementioned questions, in this study, we first determined the functional domains of MxB that are required for its anti-HCV activity. Specially, we examined whether these motifs interact with NS5A. Next, we predicted the structure of MxB in complex with NS5A through protein–protein docking method and molecular dynamic (MD) simulation. The hot-spot residues of MxB–NS5A interaction were predicted by performing binding energy decomposition calculation and validated through a series of truncation assays. Because the binding of MxB to NS5A would impair NS5A interaction with CypA, we further constructed the binary CypA–NS5A and the ternary CypA–NS5A–MxB complexes and calculated the binding affinity of CypA to NS5A in the presence or absence of MxB. Altogether, the combined theoretical and experimental study provides information on MxB–NS5A interaction and cast light on the antiviral mechanism of MxB.

## Materials and Methods

### Structure Prediction of Full-Length NS5A

The 3D structure of domain I was obtained from Protein Data Bank (PDB ID 3FQM). The 3D structure of the intrinsically disordered domains II and III was predicted using the online I-TASSER server (Roy et al., 2010; Yang and Zhang, 2015). Starting from the top 10 best structure templates identified by LOMETS (Wu and Zhang, 2007) from the PDB library, I-TASSER predicted 3D models for NS5A domains II-III via *ab initio* modeling. Out of the five predicted models, the most accurate structure had a maximum C-score (-3.04), 0.37 ± 0.13 template modeling (TM) score, and 13.3 ± 4.1 Å root mean SD (RMSD). The structure showed the highest homology with Maltose-binding periplasmic protein (PDB ID 3OSR). The predicted domain II–III model was then chosen and used as the template to create the complete structure of NS5A together with the X-ray structure of domain I (PDB ID 3FQM). Model 1 with a maximum C-score of –1.90 was chosen as the final 3D structure of full-length NS5A. The predicted structure was then embedded in a water environment and relaxed using MD simulation for 2 ns. PyMOL (version 2.4.2) was used to visualize the model and create images.

### Structure Prediction of Full-Length MxB

Our data demonstrate that the first 83 residues of MxB are critical for HCV restriction. However, no structural details about this unstructured region are available. We predicted the whole structural model of MxB by the I-TASSER server. The crystal structure of MxB (aa. 93–711, PDB ID 4WHJ) was designed as a threading template. Out of the five predicted models, model 1 with a maximum C-score of –1.75, estimated TM-score of 0.50 ± 0.15, and estimated RMSD of 12.4 ± 4.3 Å was chosen as the final 3D structure of full-length MxB. PyMOL (version 2.4.2) was used to visualize the model and create images.

### Protein–Protein Docking

The HADDOCK (version 2.2.) modeling program was used for protein–protein docking (Dominguez et al., 2003; de Vries et al., 2010). For the CypA–NS5A model, the active residues of CypA were R55, F60, and F113; the active residues of NS5A were 305–322 and 329–346. For the NS5A–MxB model, the active residues of NS5A were domain I (aa. 1–188); the active residues of MxB were 1–387 (GTPase). The passive residues were set to determine automatically. The ternary complex CypA–NS5A–MxB was predicted by using ZDOCK (Chen et al., 2003) and RDOCK (Li et al., 2003) protein–protein docking methods. The whole surface of NS5A in the NS5A–MxB complex was set as “receptors” to explore the potential binding sites of CypA.

### Molecular Dynamic Simulation

The predicted CypA–NS5A, NS5A–MxB, and CypA–NS5A–MxB complexes were embedded in water surroundings and relaxed applying MD simulations for 10 ns, respectively. All the simulations were carried out using Amber11 software with amber ff99SB force field (Lindorff-Larsen et al., 2010). The LEAP module was used to add protons and solvate CypA–NS5A, NS5A–MxB, and CypA–NS5A–MxB complexes in a box of TIP3P water, extending at least 10 Å from the complexes. To CypA–NS5A, NS5A–MxB, and CypA–NS5A–MxB complexes were added 16 Na^+^, 7 Na^+^, and 6 Na^+^ for charge neutralization, respectively.

We first minimized the positions of water and ions, while keeping the proteins fixed with a force constant of 100 kcal mol^–1^ Å^–2^. Then the entire system was minimized without any restraints. After energy minimization, the systems were heated from 0 to 300 K over 500 ps under constant volume and periodic boundary conditions (NVT). Before moving on to the production MD simulation, each system was then equilibrated with weak restrains (10 kcal mol^–1^ Å^–2^) for 500 ps at a constant pressure of 1 atm and temperature of 300 K. Finally, a length of 10-ns trajectory was computed at 300 K using an isothermal isobaric ensemble (NPT) with periodic boundary conditions. The time step was set to 2.0 fs throughout the simulation. The long-range electrostatic interactions were treated using the particle mesh Ewald (PME) method (Darden et al., 1993), and the SHAKE algorithm was applied to constrain all bonds that involved hydrogen atoms.

The trajectories and presence of hydrogen bonds were analyzed using the ptraj module in Amber. The representative structures of CypA–NS5A, NS5A–MxB, and CypA–NS5A–MxB complexes were obtained through cluster analysis by using the kclust module in MMTSB Tool Set (Feig et al., 2004). The binding free energy decomposition was calculated with molecular mechanics/generalized Born surface area (MM/GBSA) method as previously described (Hou et al., 2011; Li et al., 2021). All the simulation results were visualized using VMD (version 1.9.3).

### Plasmid DNA and Reagents

NS5A, MxB, and CypA cDNA or truncated cDNA sequences were inserted between the *Bam*HI (or *Eco*RI) and *Not*I restriction sites in the pcDNA4/TO expression vector, with Flag, Myc, and HA-tag sequences attached to the C-terminus of proteins. The antibodies used for Western blotting included mouse anti-Flag (Cat. #8146T, Cell signaling Technologies (CST), Danvers, MA, United States), rabbit anti-Flag (Cat. #14793S, GST), anti-Myc antibody (Cat. #2276S, GST), mouse anti-HA (Cat. #H3663, Sigma, St. Louis, MO, United States), and anti-beta actin (Cat. #ab8224, Abcam, Cambridge, United Kingdom).

### Cell Culture and Transfection

Huh7.5.1 cells (Rongtuan Lin, McGill University) and HEK293T cells (CRL-11268, ATCC, Manassas, VA, United States) were maintained in Dulbecco’s modified Eagle’s medium (DMEM) (Gibco, Grand Island, NY, United States) supplemented with 10% fetal bovine serum (FBS) at 37°C with 5% CO_2_. HEK293T and Huh7.5.1 cells were transfected by the use of Lipofectamine 2000 (Invitrogen, Carlsbad, CA, United States) and VigoFect (Vigorous Biotechnology, Beijing, China), respectively, in accordance with the manufacturers’ instructions.

### Western Blotting and Immunoprecipitation

HEK293T cells were transfected with plasmids expressing MxB, NS5A, CypA, or their truncated mutants for 48 h. Cells were lysed in buffer containing 25 mM of Tris, pH 7.4, 150 mM of NaCl, 1% NP-40, 1 mM of EDTA, and 5% glycerol (Pierce) on ice for 1 h and then centrifuged at 12,000 rpm for 10 min to remove cell debris. Cellular extracts were subjected to sodium dodecyl sulfate–polyacrylamide gel electrophoresis (SDS-PAGE). Proteins were transferred onto polyvinylidene difluoride (PVDF) membranes and probed with the indicated antibodies at predetermined concentrations.

For immunoprecipitation, 8% of the whole-cell lysates (8% WCL) served as input, the other lysates were incubated with indicated antibodies for 8 h, and then protein A/G agarose gel beads were added in the lysates for more than 18 h with gentle agitation at 4°C. The beads with proteins were washed with lysis buffer 3 times to remove unbound proteins, and the bound proteins were examined by Western blotting.

### Jc1 HCVcc Infections

Huh7.5.1 cells measuring 4 × 10^5^/well were seeded into 6-well plates 24 h prior to transfection with pMxB and its mutants’ plasmids. At 48 h post-transfection (hpt), cells were infected with Jc1 HCVcc at multiplicity of infection (MOI) = 1. After 72 h, cells were harvested and used for Western blotting to measure the expression level of MxB and its mutants. Amounts of Jc1 HCVcc in the supernatant were measured by determining the activity of Gaussia luciferase (Gluc), using a Centro XS3 LB 960 luminometer.

## Results

### The N-Terminal 83-aa and GTPase Domains of MxB Bind to NS5A and Are Crucial for Their Anti-HCV Activity

**FIGURE 1:**
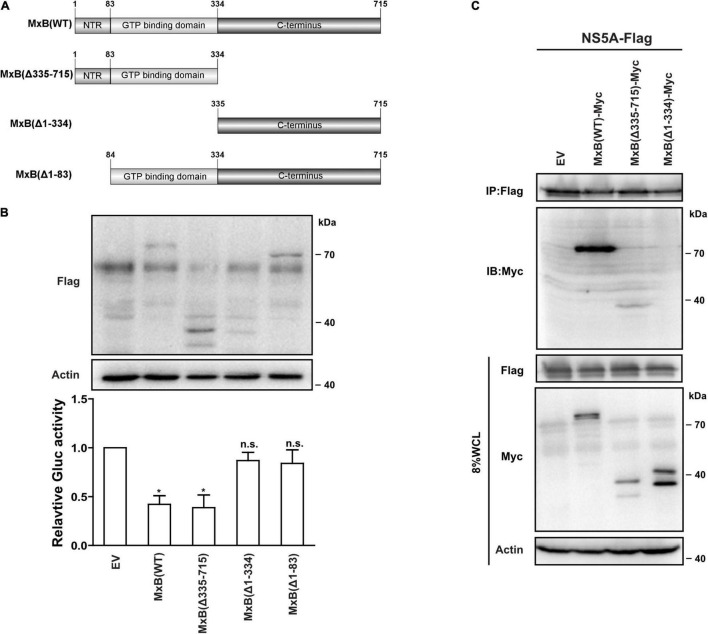
The anti-HCV activity of MxB truncations. **(A)** Schematic representation of wild-type and mutated MxB. **(B)** The anti-HCV activities of MxB truncations. Huh7.5.1 cell line was transduced with plasmids expressing Flag-tagged MxB (WT) and MxB truncations. The expression levels were examined by Western blotting analysis. The Jc1 HCVcc virus, which expresses Gluc as a reporter, was used to infect the MxB Huh7.5.1 cells. HCV infection was determined by measuring Gluc activity in the supernatant. **(C)** Co-IP was applied to detect the interaction between MxB truncations and NS5A. HEK293T cells were transfected with plasmids expressing MxB-Myc truncations and NS5A-Flag. Anti-Flag antibody was used to pull down NS5A-Flag, and anti-Myc antibody was applied to detect MxB truncations. Data are representative of three independent experiments, and values are expressed as means ± SD. Difference between two conditions is considered statistically significant with *p* < 0.05 (*). n.s. indicates non-significance. HCV, hepatitis C virus; MxB, myxovirus resistance B; WT, wild-type; Co-IP, co-immunoprecipitation.

We previously, for the first time, reported that MxB inhibits the replication of HCV through interacting with NS5A (Yi et al., 2019). However, the functional domains of MxB that are required for anti-HCV activity are not fully understood. MxB comprises an unstructured N-terminal 83-aa domain, a GTP binding region (aa. 84–334) (Melen et al., 1996), and the remaining C-terminal structures including BSE and stalk regions (aa. 335–715). To determine the functional domains of MxB that were related to its anti-HCV activity, we produced three truncated MxB proteins (Figure 1A): MxB(Δ1–83), MxB(Δ1–334), and MxB(Δ335–715). Then we examined the anti-HCV activities of MxB mutants by transfecting Huh7.5.1 cells with a vector carrying the wild-type (WT) and mutated MxB cDNA, followed by infection with Jc1 HCVcc virus, which expresses Gluc as a reporter. As seen in Figure 1B, the expression levels of these truncated MxB proteins were similar to those of MxB (WT). Interestingly, MxB (Δ1–83) and MxB(Δ1–334) ablated the anti-HCV activity of MxB. In construct, when the first 334 residues are retained, the antiviral activity of MxB(Δ335–715) was equivalent to that of WT MxB. These results further confirmed the crucial role of the N-terminal 83-aa domain and GTP binding domain of MxB in its antiviral function.

We previously reported that MxB exhibited its anti-HCV activity through interacting with NS5A. To investigate the binding region of MxB, we determined the protein–protein interaction between NS5A and MxB truncations using the co-immunoprecipitation (co-IP) technique. A Myc-MxB/Flag-NS5A co-complex was detected (Figure 1C, lane 2), confirming the complex of MxB–NS5A. The interaction between MxB and NS5A was lost when the first 334 residues were removed (Figure 1C, lane 4). Taken together, the first 334 residues of MxB, including the N-terminal 83-aa domain and GTP binding region, bind to NS5A and are crucial for the anti-HCV activity of MxB.

### The Protein–Protein Interaction Between MxB and NS5A

#### Predicted Complex Structure of MxB–NS5A

We previously reported that MxB exhibited its anti-HCV activity through interacting with NS5A-D1 (Yi et al., 2019). However, the molecular details of the MxB–NS5A binding interface are not clear. Here we predicted the MxB–NS5A interaction hot-spot amino acids using computational methods and validated the key interactions through truncation assays.

Since no structural details about the highly disordered NS5A-D2D3 domains and the N-terminal 83-aa domain of MxB are currently available, we first predicted the 3D structure of these regions by the I-TASSER server via *ab initio* modeling prior to molecular docking studies. The predicted full-length structure of NS5A and MxB is shown in Supplementary Figure 1. Then we utilized protein–protein docking program HADDOCK2.2 to predict the MxB–NS5A complex structure. The N-terminal 83-aa and GTPase domains of MxB and NS5A-D1 were set as active binding regions. As a result, a total of 163 structures in 9 clusters were generated. Among them, model 1 with the lowest *Z*-score of − 2.5 was chosen as the final 3D structure of the MxB–NS5A complex. This binary complex was then embedded in a water environment and relaxed using MD simulations for 10 ns. Trajectory analysis indicates that the MxB–NS5A complex structure remained stable during MD simulations (Supplementary Figure 2).

**FIGURE 2:**
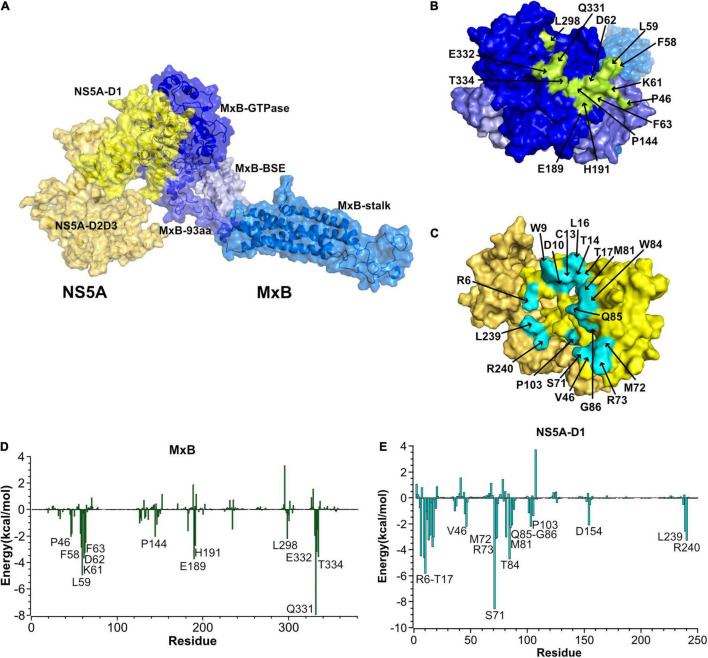
Predicted MxB–NS5A interaction sites. **(A)** Cartoon and surface representation of the most representative structure of MxB (blue) in complex with NS5A (yellow). NS5A uses its domains I (NS5A-D1) to bind to the GTPase domain of MxB. **(B,C)** Surface representations of interacting residues in MxB (top) and NS5A (bottom). The identified hot-spot residues in MxB and NS5A are labeled and highlighted in green and blue in the surface representations, respectively. **(D,E)** The per-residue contribution to the MxB:NS5A-D1 binding energy (kcal/mol). Only residues with total energies above 2 kcal/mol are labeled. NS5A, non-structural protein 5A; MxB, myxovirus resistance B.

Then we performed a cluster analysis to select the most representative structure of the MxB–NS5A complex. As depicted in Figure 2A, MxB mainly uses its N-terminal domain and GTPase domain to bind to the head of NS5A-D1. The contact residues were heightened in Figures 2B,C. The per-residue contribution to the MxB–NS5A binding energy is depicted in Figures 2D,E. As seen in Figures 2B,D, six residues (P46, F58, L59, K61, D62, and F63) in the N-terminal 83-aa domain of MxB are involved in the binding of NS5A. In the GTPase domain of MxB, P144, E189, H191, L298, Q331, E332, and T334 contribute the majority of the binding energies with the NS5A-D1.

As seen in Figures 2C,E, the hydrophobic cavity of NS5A consists of residues W9, T14, L16, T17, M72, M81, W84, P103, and L239, while the other residues, such as R6, D10, C13, V46, S71, R73, Q85, G86, and R240, form hydrophilic interaction with MxB. Among them, the oxygen atom of NS5A-G86 forms an H-bond with the side-chain nitrogen atoms of MxB-Q331. The average bond length of this H-bond is 2.8 Å, and the percentage occupation is 84.8%. Besides, NS5A residues R6, S71, and K101 form hydrogen bonds with MxB residues E189, E332, and T334, respectively. All hydrogen bonds discussed here are available in Table 1. These H-bond networks can facilitate the stabilization of the MxB–NS5A complex.

**TABLE 1:** H-bond analysis for MxB:NS5A protein–protein interaction.

MxB	NS5A	Percentage occupancy (%)	Average distance (Å)
Q331 [NE2]	G86 [O]	84.8	2.8
E332 [OE2]	S71 [OG]	79.0	2.7
T334 [OG1]	K101 [O]	63.4	2.8
E189 [OE2]	R6 [NH2]	52.2	2.8
E189 [OE1]	R6 [NH1]	50.6	2.8
Q331 [NE2]	M72 [O]	48.6	2.9
S132 [OG]	Q85 [OE1]	36.6	2.7
D297 [O]	R73 [NE]	33.6	2.9

*MxB, myxovirus resistance B; NS5A, non-structural protein 5A.*

#### Validation of the Predicted MxB–NS5A Interaction

**FIGURE 3:**
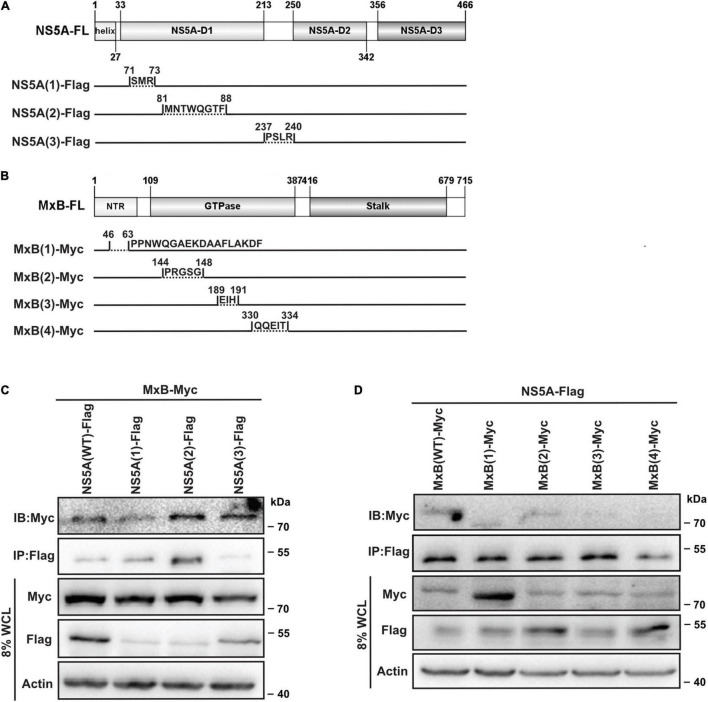
Validation of the predicted MxB–NS5A interaction. **(A,B)** Schematic representation of NS5A and MxB truncations. Diagrams were constructed using IBS (version 1.0) (Liu et al., 2015). The interactions of MxB with NS5A truncations **(C)** and those of NS5A with MxB truncations **(D)** were detected by IP with anti-Flag antibody followed by immunoblotting (IB) with anti-Myc antibody in HEK293T. Data are representative of three independent experiments. MxB, myxovirus resistance B; NS5A, non-structural protein 5A; IP, immunoprecipitation.

To verify the predicted interactions between MxB and NS5A, we constructed a series of NS5A and MxB deletion mutants, respectively (Figures 3A,B). Then we examined whether these mutants could associate with the target proteins through co-IP experiments. Full-length Myc-MxB and Flag-NS5A (WT and truncations) were co-expressed in HEK293T cells. The association of MxB with NS5A (WT and truncations) was determined by IP with anti-Flag antibody followed by immunoblotting (IB) with anti-Myc antibody. As seen in Figure 3C, the Myc-MxB/Flag-NS5A co-complex was detected, confirming the existence of the MxB–NS5A complex (Figure 3C, lane 1). Weakened interaction was observed when the N-terminal domain (aa. 71–73) of NS5A was removed (Figure 3C, lane 2), whereas deletion of aa. 81–88 or aa. 237–240 of NS5A did not affect the MxB–NS5A interaction (Figure 3C, lanes 3, 4).

To detect whether the predicted motif in the N-terminal domain and GTP binding domain of MxB allows protein–protein interaction, co-IP assays were performed with full-length Flag-NS5A and Myc-MxB (WT and truncations) (Figure 3D). Protein–protein interactions were examined by IP with anti-Flag antibody followed by IB with anti-Myc antibody in HEK293T cells. The results showed that removal of the GTP binding domain residues aa. 189–191 or aa. 330–334 completely abrogated the MxB–NS5A interaction (Figure 3D, lanes 4, 5).

Combined, these results showed that aa. 71–73 in NS5A-D1 and MxB GTP binding domain residues aa. 189–191 and aa. 330–334 are crucial for MxB–NS5A interaction.

### The Protein–Protein Interaction Between CypA and NS5A

We previously reported that the binding of MxB to NS5A would impair the interaction between NS5A and CypA (Yi et al., 2019). To examine this inhibition mechanism at the atomistic level, we also predicted the CypA–NS5A complex structure and validated the model through truncation assays.

#### Predicted Complex Structure of CypA–NS5A

**FIGURE 4:**
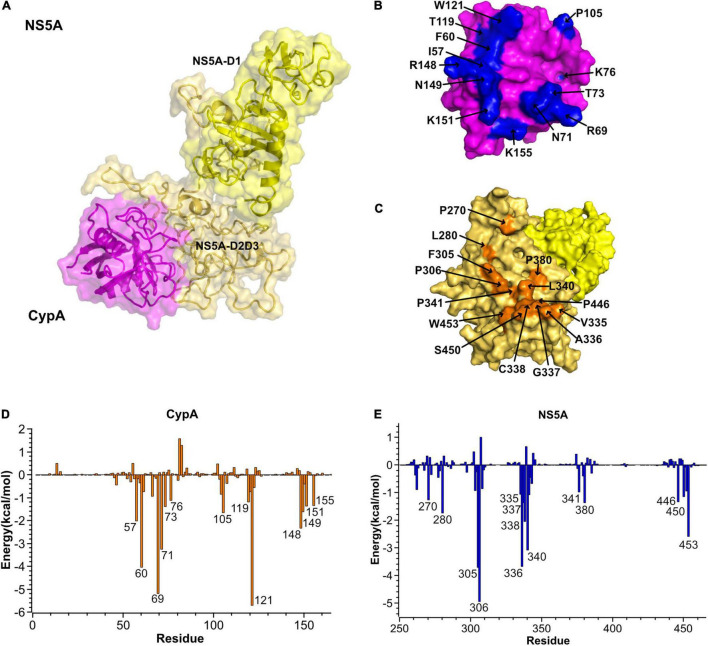
Predicted CypA–NS5A interaction sites. **(A)** Cartoon and surface representation of the most representative structure of CypA (magenta) in complex with NS5A (yellow). NS5A uses its domain I and domain II (NS5A-D2D3) to bind CypA. **(B,C)** Surface representations of interacting residues in CypA (top) and NS5A (bottom). The identified hot-spot residues in CypA and NS5A are labeled and highlighted in blue and orange in the surface representations, respectively. **(D,E)** The per-residue contribution to the CyPA:NS5A binding energy (kcal/mol). Only residues with total energies above 1 kcal/mol are labeled. CypA, cyclophilin A; NS5A, non-structural protein 5A.

Spot-binding assay for the CypA–NS5A interaction identified that CypA interacts with the proline-rich regions of NS5A-D2D3 (Grise et al., 2012; Ngure et al., 2016). Among them, P310 and P341 were required for CypA binding. Besides, mutations R55A, F60A, and F113A of CypA completely or largely abolished NS5A binding (Yang et al., 2010; Foster et al., 2011). On the basis of the experimental data, we predicted the CypA–NS5A complex by the protein–protein docking program HADDOCK2.2. The residues mentioned above were defined as binding sites during the docking process. CypA binds to the predefined sites and was anchored by NS5A–D2. The complex was then relaxed using MD simulations for 10 ns. Trajectory analysis indicates that the relative orientation of CypA to NS5A did not change during simulation (Supplementary Figure 2). The most representative structure was depicted in Figure 4A.

The predicted complex structure reveals that NS5A mainly uses its proline-rich domain D2 to engage a hydrophobic pocket of CypA. The contact residues are highlighted in Figures 4B,C. The per-residue contribution to the CypA: NS5A binding energy was depicted in Figures 4D,E. In NS5A-D2, 14 residues (P270, L280, F305, P306, V335, A336, G337, C338, L340, P341, P380, P446, S450, and W453) are involved in the interface. Among the residues, F305, P306, A336, and L340 contribute the majority of the binding energies and form tight hydrophobic interaction with CypA. Correspondingly, the hydrophobic cavity of CypA consists of residues I57, F60, W73, P105, and W121, while the other residues, such as R69, N71, K76, T119, R148, N149, K151, and K155, form a hydrophilic patch.

Then we examined the hydrogen bond networks in CypA–NS5A complex. The oxygen atom of NS5A-S303 forms an H-bond interaction with the side-chain nitrogen atoms of CypA-N71. The percentage occupation of this H-bond is 63.4% during 10-ns MD, and the average bond length is 2.8 Å. Besides, residues E262, S303, and S450 form hydrogen bonds with R69, N71, and 59 of CypA in the cavity, respectively. All hydrogen bonds discussed here are available in Table 2.

**TABLE 2:** H-bond analysis for CyPA:NS5A protein–protein interaction.

NS5A	CypA	Percentage occupancy (%)	Average distance (Å)
S303 [O]	N71 [ND2]	63.4	2.8
E262 [OE2]	R69 [NE]	47.0	2.8
S450 [OG]	G59 [O]	39.6	2.7
S452 [O]	R148 [NH1]	39.2	2.8
E262 [OE2]	R69 [NH2]	39.0	2.8

*CypA, cyclophilin A; NS5A, non-structural protein 5A.*

#### Validation of the Predicted CypA–NS5A Interaction

**FIGURE 5:**
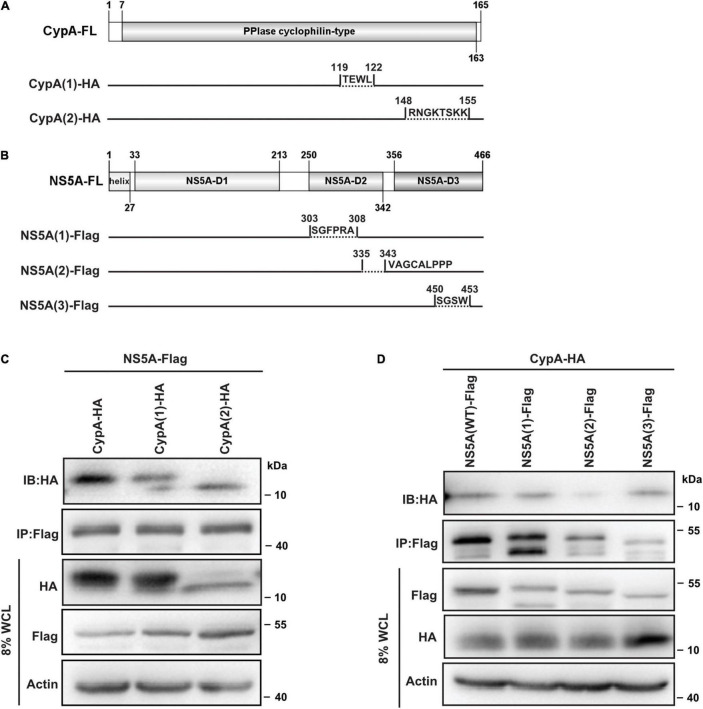
Validation of the predicted CypA–NS5A interaction. **(A,B)** Schematic representation of CypA and NS5A truncations. Diagrams were constructed using IBS (version 1.0) (Liu et al., 2015). The interactions of NS5A with CypA truncations **(C)** and that of CypA with NS5A truncations **(D)** were detected by IP with anti-Flag antibody followed by immunoblotting (IB) with anti-HA antibody in HEK293T. Data are representative of three independent experiments. CypA, cyclophilin A; NS5A, non-structural protein 5A; IP, immunoprecipitation.

To verify the predicted interactions between CypA and NS5A, we constructed a series of NS5A and CypA deletion mutants, respectively (Figures 5A,B). Then we examined whether these mutants could associate with the target proteins through co-IP experiments.

To monitor the key residues identified in CypA, we co-expressed full-length Flag-NS5A and HA-CypA (WT and truncations) in HEK293T cells. The association of NS5A with CypA (WT) or CypA deletion mutants was determined by IP with anti-Flag antibody followed by IB with anti-HA antibody. The HA-CypA/Flag-NS5A co-complex was detected, confirming the existence of the CypA–NS5A complex (Figure 5C, lane 1). The interaction between CypA and NS5A was weakened when the aa. 119–122 or aa. 148–155 of CypA were removed (Figure 5C, lanes 2, 3).

To detect whether the predicted motif in the NS5A-D2D3 allows protein–protein interaction, co-IP assays were performed with full-length HA-CypA and Flag-NS5A (WT and truncations). An HA-CypA/Flag-NS5A co-complex was detected (Figure 5D, lane 1), confirming the existence of the CypA–NS5A complex. Removal of the NS5A-D2 residues aa. 335–343 greatly reduced the binding of NS5A to CypA (Figure 5D, lane 3).

Taken together, these results show that amino acids aa. 119–122 and aa. 148–155 in CypA and NS5A-D2 aa. 335–343 are crucial for CypA–NS5A interaction.

### Potential Ternary CypA–NS5A–MxB Complex

Collectively, MxB and CypA bind to NS5A-D1 and NS5A-D2D3, respectively. Given that the binding of MxB to NS5A would impair the CypA–NS5A interaction, we constructed the ternary CypA–NS5A–MxB complex and compared binding energy changes of CypA–NS5A in the presence or absence of MxB.

**FIGURE 6:**
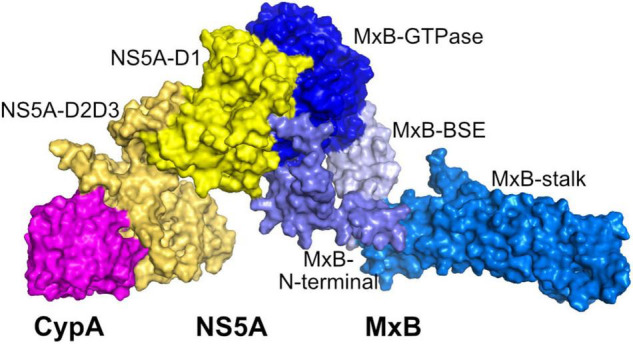
Predicted ternary CyPA–NS5A–MxB complex structure. CyPA, NS5A, and MxB are shown in magenta, yellow, and blue surface, respectively. CypA, cyclophilin A; NS5A, non-structural protein 5A; MxB, myxovirus resistance B.

We first predicted the ternary CypA–NS5A–MxB complex by docking CypA to the binary complex NS5A–MxB using HADDOCK to yield starting conformation. The initial structure was then simulated for 10 ns, and the RMSD plot shows that the structures are stable during the course of MD simulations (Supplementary Figure 2). As shown in Figure 6, MxB and CypA bind to different domains of NS5A, and the bindings sites are spaced far from each other. It seems that the binding of MxB to the NS5A-D1 region would not block the CypA-binding region.

Then we calculated the binding energies of CypA to NS5A in the presence or absence of MxB. As seen in Table 3, the calculated MM-GBSA binding energies of MxB to NS5A (−105.33 kcal/mol) are much lower than those of CypA (−61.57 kcal/mol), which means that MxB shows stronger binding affinity than CypA. Most importantly, a 28% decrease in CypA binding affinity was observed with the existence of MxB. The MM-GBSA binding energy increased from − 61.57 to − 44.14 kcal/mol. These results suggested that MxB could play a role in the energetic destabilization of CypA–NS5A binding.

**TABLE 3:** The MM-GBSA binding energy of the binary complexes (CyPA:NS5A; MxB:NS5A) and ternary complex (CyPA:NS5A–MxB).

Complex	Time (ps)	Average binding energy (kcal/mol)	Std error of the mean (kcal/mol)
CyPA:NS5A	10,000	-61.57	1.03
MxB:NS5A	10,000	-105.33	1.99
CyPA:NS5A–MxB	10,000	-44.14	1.37

*CypA, cyclophilin A; NS5A, non-structural protein 5A; MxB, myxovirus resistance B.*

**FIGURE 7:**
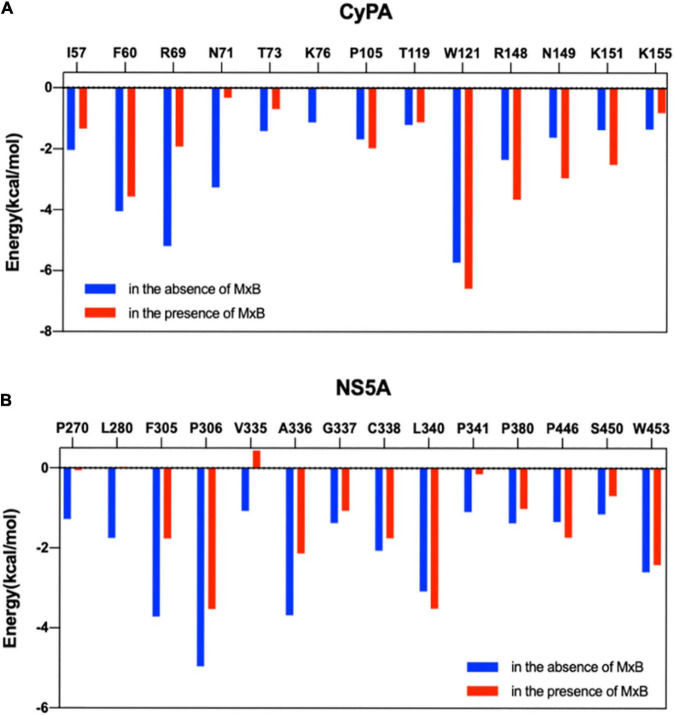
Comparison of per-residue energy decomposition for key residues of CyPA:NS5A interaction in the absence (blue) and presence (red) of MxB. Residues highlighted are displayed for CyPA **(A)** and NS5A **(B)**. CypA, cyclophilin A; NS5A, non-structural protein 5A; MxB, myxovirus resistance B.

Furthermore, MM/GBSA per-residue energy decomposition analysis was employed to investigate the energetic contribution of hot-spot residues at the CypA–NS5A interface in the presence of MxB. As illustrated in Figure 4, 13 residues in CypA and 14 residues in NS5A were identified as key residues for CypA–NS5A interaction. Figure 7A reveals that the energetic contribution of I57, F60, R69, N71, T73, K76, and K155 in CypA decreased in the presence of MxB. Particularly, R69 in CypA contributed − 5.19 and − 1.91 kcal/mol in the absence and presence of MxB, respectively. The absolute binding energy of N71 also greatly decreased from 3.25 to 0.32 kcal/mol.

Figure 7B revealed that the absolute binding energy values of 12 out of 14 residues in NS5A decreased in the presence of MxB. Particularly, aa. 335–343 were verified as key residues for the binding of NS5A to CypA (Figure 5C). P341 of NS5A has been reported to be required for CypA function.

## Discussion

The human antiviral protein MxB conveys host resistance to a variety of infectious viruses (Goujon et al., 2013; Haller, 2013; Liu et al., 2013; Crameri et al., 2018; Steiner and Pavlovic, 2020; Wang et al., 2020). Previously, we have identified MxB as a key factor behind IFN-mediated suppression of HCV infection, greatly expanding the antiviral spectrum of MxB (Yi et al., 2019). Data in this study further characterized the functional domains in MxB that are crucial for HCV restriction. Specifically, we found that the N-terminal 83-aa domain and GTP binding domain are indispensable for the anti-HCV activity of MxB, with deletion of these regions ablating the inhibitory effects (Figure 1B). The N-terminal 83-aa, especially the GTPase domain, has been thoroughly studied and identified to be critical for the inhibition of HIV-1 and herpesviruses (Lemke et al., 2013; Fribourgh et al., 2014; Goujon et al., 2014). Interestingly, the stalk region, which is critical for MxB oligomerization, had no significant effects on the anti-HCV activities. Previous studies reported that the stalk region is critical for MxB oligomerization (Alvarez et al., 2017), which is required for the ability of MxB to bind to the HIV-1 core and block HIV-1. However, we observed that the anti-HCV activity of stalk domain truncated (Δ335–715) MxB protein was equivalent to that of WT MxB (Figure 1B). The detailed role of the stalk region for the antiviral function of MxB warrants further study.

The interactions between host-encoded and virus-encoded proteins play critical roles in viral replication (Chigbu et al., 2019). We reported earlier that host cellular protein MxB was specifically associated with HCV-encoded NS5A and did not interact with other HCV proteins (Yi et al., 2019). Data in this study further confirmed that MxB residues aa. 189–191 and aa. 330–334, together with NS5A residues aa. 71–73, are essential for the overall binding of the two proteins (Figure 3). Most importantly, NS5A-S71 forms strong H-bond interaction with MxB-Q331, which leads to a stabilization of the NS5A–MxB complex (Table 1). This host–virus interaction will diminish the binding of NS5A to another host protein CypA and thus block HCV replication. Previous experimental studies illustrated that MxB and CypA bind to the NS5A-D1 and NS5A-D2D3, respectively (Hanoulle et al., 2009; Foster et al., 2011; Verdegem et al., 2011; Ngure et al., 2016). Consistent with this observation, in this study, we observed that MxB and CypA bind to a different region of NS5A, and the binding sites are far from each other (Figure 6). Most importantly, a 28% decrease in CypA–NS5A binding affinity was predicted in the existence of MxB (Table 3), suggesting that MxB plays a role in the energetic destabilization of CypA–NS5A binding. Specifically, the absolute binding energy of key residues R69 and N71 in CypA, together with V335, A336, and P341 in NS5A, greatly decreased in the presence of MxB (Figure 7). These results propose potential binding modes of binary CypA–NS5A and NS5A–MxB complexes but are not able to eliminate the possibility that MxB exhibits its anti-HCV activity by other biologically active factors.

Our study also has some limitations. Before starting the complex assembly via docking, we had to build the structures of the full-length MxB and NS5A proteins because these structures are not available in experimental databases. However, both the MxB N-terminal 83-aa domain and NS5A-D2D3 region are intrinsically disordered. It was difficult to obtain convincing confirmation of these regions using protein structure prediction software. Perhaps the AlphaFold2 program could be used to create these starting structures in the future study.

## Conclusion

Investigations of protein–protein interactions involving MxB, NS5A, and CypA at the molecular level facilitate the understanding of the anti-HCV activity of MxB. In this study, we first characterized the functional domains of MxB that are crucial for its anti-HCV activity. Subsequently, a combined computational and experimental approach was performed to examine the binding interface of MxB–NS5A, CypA–NS5A, and CypA–NS5A–MxB. Binding energy calculation highlighted that the binding of MxB to NS5A will decrease the binding affinity of CypA–NS5A, thus inhibiting HCV replication. We hope this work would provide a possible explanation for the understanding of the antiviral activity of MxB and may provide clues for designing new strategies to combat CypA-dependent viruses.

## Data Availability Statement

The original contributions presented in the study are included in the article/Supplementary Material, further inquiries can be directed to the corresponding author/s.

## Author Contributions

SC conceptualized the study and supervised the study. DY and QL did the formal analysis and carried out the investigation. XY and NA performed the co-IP experiments. HS and RZ prepared the protein samples. QL wrote the original draft. SC and DY reviewed and edited the manuscript. All authors contributed to the article and approved the submitted version.

## Conflict of Interest

The authors declare that the research was conducted in the absence of any commercial or financial relationships that could be construed as a potential conflict of interest.

## Publisher’s Note

All claims expressed in this article are solely those of the authors and do not necessarily represent those of their affiliated organizations, or those of the publisher, the editors and the reviewers. Any product that may be evaluated in this article, or claim that may be made by its manufacturer, is not guaranteed or endorsed by the publisher.
