# Supplementary material for: MxB Disrupts Hepatitis C Virus NS5A–CypA Complex: Insights From a Combined Theoretical and Experimental Approach
Source: Front Microbiol. 2022 Mar 17;13:849084. doi: 10.3389/fmicb.2022.849084 (PMC8969595; doi:10.3389/fmicb.2022.849084)
Supplement: Supplementary file 1 [file Data_Sheet_1.PDF]

Supporting Information

MxB disrupts Hepatitis C virus NS5A–CypA complex: Insights from a combined theoretical and experimental approach

Quanjie Li[1,#], Ni An[1,#], Xiao Yin[1,2], Ruixin Zhang[1], Huihan Shao[1], DongrongYi [1,*], Shan Cen [1,*]

[1]Department of Immunology, Institute of Medicinal Biotechnology, Chinese Academy of Medical Sciences, Beijing 100050, China.

[2]Center for drug evaluation, National Medical Products Administration, Beijing 100037, China.

*** Correspondence:**

E-mail addresses: dongrong.yi@imb.pumc.edu.cn (D.Y.); shancen@imb.pumc.edu.cn (S.C.).

[#] These authors contributed equally to this work.

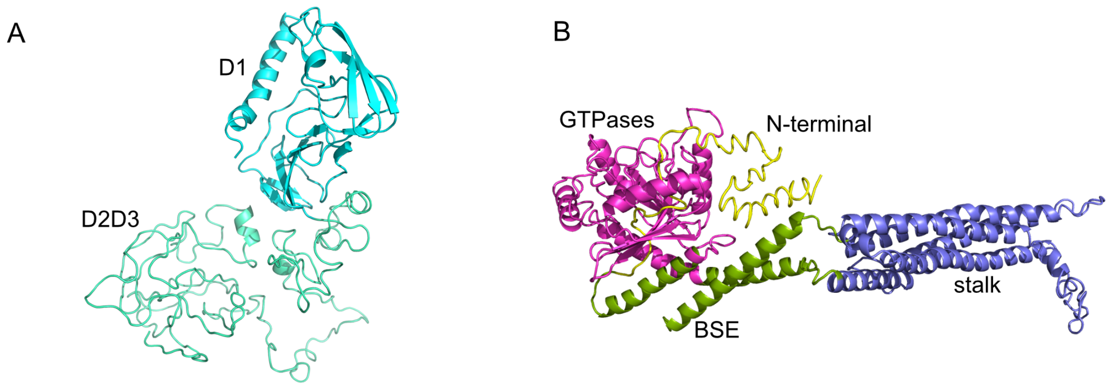

Figure S1. Predicted full-length structure of NS5A**(A)** and MxB**(B)**.

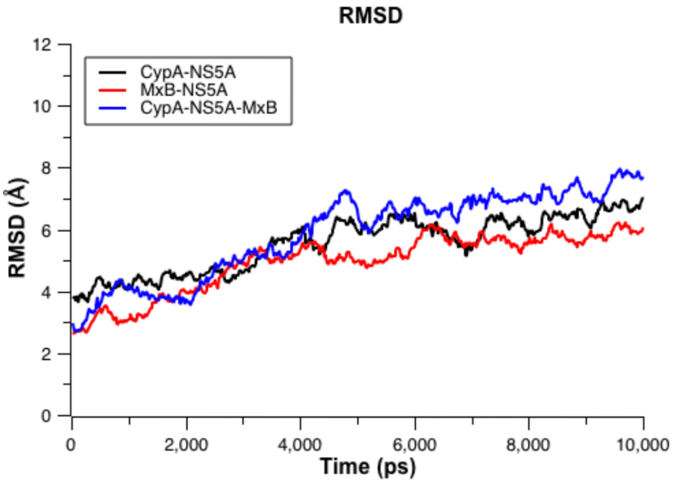

Figure S2. Root-mean square deviation (RMSD) values for the backbone atoms. Black, red, and blue lines represent complexes of CypA–NS5A, MxB–NS5A, and CypA–NS5A–MxB, respectively.
